# Editorial: Probiotics for global health: advances, applications and challenges

**DOI:** 10.3389/fmicb.2025.1697319

**Published:** 2025-10-15

**Authors:** Karthika Bahuleyan Arun, Prakasan Nisha, Stavros Plessas, Natasa Golic, Astghik Pepoyan

**Affiliations:** ^1^Department of Life Sciences, CHRIST (Deemed to be University), Bangalore, Karnataka, India; ^2^Agro Processing and Technology Division, CSIR-National Institute for Interdisciplinary Science and Technology, Council of Scientific and Industrial Research, Trivandrum, India; ^3^Academy of Scientific and Innovative Research (AcSIR), Ghaziabad, India; ^4^Laboratory of Food Microbiology, Biotechnology, and Hygiene, Department of Agriculture Development, Democritus University of Thrace, Orestiada, Greece; ^5^Institute of Molecular Genetics and Genetic Engineering, University of Belgrade, Belgrade, Serbia; ^6^Food Safety and Biotechnology Department, Scientific Research Institute of Food Science and Biotechnology, Armenian National Agrarian University, Yerevan, Armenia; ^7^International Association for Human and Animals Health Improvement, Yerevan, Armenia

**Keywords:** probiotic, gut microbiota, immune modulation, metabolic health, gut–brain axis, antimicrobial activity

## Introduction

In recent decades, probiotics have become a central focus in biomedical and nutritional sciences due to their ability to support host health, prevent disease, and counteract dysbiosis. Given the rising global burden of disease, there is an urgent need for safe, sustainable, and accessible interventions that can complement conventional therapies. Probiotics, defined as live microorganisms that confer a health benefit when administered in adequate amounts, represent a promising strategy for improving public health across diverse populations and life stages.

This Research Topic was conceived to examine the role of probiotics in advancing global health and contributing to the United Nations Sustainable Development Goal 3 (SDG3): “Ensure healthy lives and promote wellbeing for all at all ages.” The contributions published here highlight the multifaceted impact of probiotics on human wellbeing, with topics spanning infectious disease prevention, chronic condition management, maternal and infant health, mental health, and antimicrobial resistance mitigation.

A consistent theme is the ability of probiotics to reduce pathogen colonization, enhance mucosal defenses, and modulate immune responses, offering cost-effective approaches to alleviating infectious disease in vulnerable populations. Probiotics also help manage non-communicable diseases such as cancer, cardiovascular disorders, diabetes, and obesity by modulating metabolism, reducing inflammation, and strengthening the gut barrier. Probiotic interventions also support maternal health and infant development, while early-life supplementation can reduce the risk of neonatal infections, allergies, and gastrointestinal disorders.

Probiotics are being increasingly studied for their impact on the gut–brain axis, including their effect on neurotransmitters, neuroinflammation, and stress. Finally, their contribution to combating antimicrobial resistance (AR) emphasizes their global relevance for both medicine and agriculture.

Taken together, the articles in this Research Topic synthesize recent advances in probiotic science, highlighting opportunities and challenges and underscoring their potential to transform preventive health strategies and therapeutic interventions in line with global health priorities.

## Taxonomic trends in the collected articles

The current Research Topic brought together 31 unique contributions, collectively illustrating the breadth and diversification of probiotic research (Table 1). A clear taxonomic pattern emerges, reflecting both the maturity of classical probiotic investigations and the growing interest in non-traditional microbial candidates.

**Table 1:** Taxonomic distribution and functional roles of probiotics and related interventions in the Research Topic.

**Taxonomic group/intervention**	**Number of articles**	**Functional effects**	**Target disease/health Area**	**Representative studies**
**Lactobacilli** (incl. *Lacticaseibacillus, Limosilactobacillus, Lactiplantibacillus*)	12	Immunomodulation, antioxidant activity, gut barrier reinforcement, pathogen exclusion, and metabolite production	Gastrointestinal health, allergy, oxidative stress, oncology, and pathogen control	(Zhang et al.; Wang et al.; Kiousi et al.; Dong et al.)
**Bifidobacteria**	2	Gut barrier integrity, immune modulation, pathogen antagonism, and microbiota modulation	Pediatric health, IBD, gut–brain axis	Ma et al.; Sarita et al.
***Bacillus*****/Spore-formers spp**.	1	Stress resistance, anti-inflammatory, antioxidant, and metabolic regulation	Hyperuricemia, metabolic disorders	Kallur et al.
**Next-generation probiotics (e.g., Akkermansia**, ***Faecalibacterium*****)**	2	Gut barrier maintenance, metabolic modulation, and SCFA-driven immune balance	Metabolic disorders, obesity, and inflammation	(Lu et al.; Song et al.; Sitdhipol et al.)
**Emerging genera (e.g.**, ***Blautia***, ***Enterococcus***, ***Weizmannella***, ***Aspergillus*****)**	4	SCFA production, antimicrobial activity, antioxidant and anti-inflammatory effects	Intestinal inflammation, pathogen control, metabolic disturbances	(Chen et al.; Li et al.; Gao et al; Seidler et al.)
**Synbiotic/Multistrain formulations**	3	SCFA production, microbiota diversity restoration, and inflammation reduction	Gastrointestinal disorders, metabolic health	(Liao et al.; Teng et al.; Ma et al.)
**Non-probiotic/Dietary modulators**	2	Microbiota modulation, SCFA enhancement, immunomodulation, and anti-biofilm activity	Aging-related gut health, infection, and metabolic regulation	(Deng et al.; Chowdhury et al.)
**Microbiome-linked metabolic factors**	1	Modulation of TMAO levels and regulation of cardiovascular and metabolic risk	Cardiovascular and metabolic disease	(Yu et al.)

The table provides a comprehensive overview of the thematic distribution within the field of probiotic research and should not be interpreted as a direct quantitative count of the articles.

SCFA, Short-chain fatty acids; IBD, Inflammatory bowel disease; TMAO, Trimethylamine-N-oxide.

The Lactobacillus lineage, including *Lacticaseibacillus, Limosilactobacillus*, and *Lactiplantibacillus* species, was by far the most represented group. Twelve studies, presented by Wang et al., Kiousi et al., Dong et al., and Zhang et al., accounted for almost 39% of the collected works. This predominance reflects the long-standing GRAS status of lactobacilli, their resilience in gastrointestinal environments, their immunomodulatory mechanisms, and their wide availability in food and pharmaceutical markets.

In contrast, only two articles specifically addressed the genus *Bifidobacterium* (Ma et al.; Sarita et al.), one of which is a general review covering multiple probiotics (Sarita et al.). Despite the genus's central role in early-life microbiota, immune development, and pediatric health, this limited representation underscores both a gap in research and an opportunity to further explore bifidobacterial strains in clinical and nutritional contexts.

Another single study was dedicated to *Bacillus* spore-formers, specifically *Bacillus coagulans* (Kallur et al.). With its exceptional resistance to heat, acidity, and processing stress, *B. coagulans* is gaining recognition as a robust probiotic candidate for scalable food and nutraceutical applications.

In addition, next-generation probiotics received initial but promising attention. One article examined *Akkermansia muciniphila* (Lu et al.), reflecting the field's gradual shift toward precision microbiome modulation and individualized interventions for metabolic and inflammatory disorders.

Finally, eight articles explored non-traditional and emerging taxa, including *Enterococcus, Blautia*, and *Weizmannella*, along with fungal candidates such as *Aspergillus*. Examples include the characterization of *Blautia producta* for its anti-inflammatory effects (Chen et al.) and *Enterococcus casseliflavus* for its safety profile and immunoregulatory potential (Li et al.). These contributions highlight the expanding search for alternative probiotics beyond the traditional lactobacilli and bifidobacteria.

Taken together, the taxonomic distribution across this Research Topic reveals a dual narrative: the continued centrality of lactobacillus as a model probiotic on the one hand, and the diversification of microbial candidates that may offer novel solutions to global health challenges on the other. This balance underscores how probiotic science is simultaneously building on established foundations while opening to innovation and expansion into underexplored taxa.

**Lactobacilli**

A significant portion of recent studies in this Research Topic focused on the diverse functional roles of lactobacilli, reaffirming their centrality in probiotic science and their evolution into models for next-generation functional and therapeutic interventions.

For example, *L. reuteri* demonstrated immunomodulatory effects in allergic diseases by restoring Treg/Th17 balance and by identifying luteolin as a key anti-inflammatory metabolite (Zhang et al.). Genomic and safety evaluations of *Lcb. paracasei* and *Lcb. casei* confirmed the absence of virulence and AR genes, supporting their use in food and nutraceutical applications (Chen et al.). Similarly, *Lpb. plantarum* L19 exhibits strain-specific antioxidant and stress-resistance traits, highlighting its potential to mitigate oxidative stress, for example, under heat stress in livestock (Wang et al.). Comprehensive analyses of *Lcb. paracasei* LC86 and *Lcb. casei* LC89 further confirmed their safety through genomic and phenotypic assessments and *in vivo* acute toxicity studies (Chen et al.).

Beyond gastrointestinal health, lactobacilli showed the potential to absorb microplastics and reduce intestinal accumulation and inflammation (Teng et al.), and to reinforce the host–pathogen interface by reducing *Staphylococcus aureus* and *Escherichia coli* adhesion and epithelial cell death (Kiousi et al.). Encapsulation of probiotics and synbiotics was highlighted as a strategy to enhance survival rates and expand applications in immune, metabolic, and neurological health (Sarita et al.). Mechanistic studies on *Lacticaseibacillus rhamnosus* LRa05 showed modulation of cytokines, oxidative stress, and gut microbiota, while engineering of *Lcb. paracasei* EG005 to enhance superoxide dismutase activity illustrated the potential of precision probiotics with tailored antioxidant capacity (Dong et al.; Kim et al.).

Other strains demonstrated targeted health effects, including prevention of constipation via microbiota modulation by *Lcb. rhamnosus* Glory LG12 (Ma et al.), biofilm formation and antioxidant activity of *Ligilactobacillus salivarius* LS-ARS2 (Patra et al.), and mitigation of heat stress in dairy cows by *Lpb. plantarum* L19 (Wang et al.). *Lpb. plantarum* strains ONU 12 and ONU 355, along with *Lcb. casei* ATCC 393 inhibited hepatocellular carcinoma and cholangiocarcinoma cell proliferation, synergized with chemotherapeutics, and induced apoptosis and senescence (Duduyemi et al.). *Lcb. casei* KACC92338 exhibited antioxidant, stress tolerance, and antimicrobial properties with genomic safety, highlighting its probiotic potential (Kandasamy et al.).

These studies confirm the central role of lactobacilli while showing their expanding applications, from allergy and oncology to environmental health, within the broader One Health framework.

## Bifidobacteria

The genus *Bifidobacterium* is a cornerstone of the gut microbiota in early life and remains a central focus in probiotic research due to its strain-specific roles in maintaining intestinal and systemic health. Among the reviewed studies, *Bifidobacterium* spp. demonstrated key physiological effects, including enhancement of mucosal barrier integrity, immune modulation, and antagonism toward pathogenic microbes. These studies highlighted the genus's diverse therapeutic potential, ranging from the alleviation of metabolic and neuroinflammatory markers to the improvement of inflammatory bowel disease when *Bifidobacterium animalis* subsp. *lactis* XLTG11 was combined with mesalazine, resulting in superior anti-inflammatory and microbiota-modulating effects compared to either treatment alone. Furthermore, several articles explored the synergistic effects of combining *Bifidobacterium* spp. with other probiotics/prebiotics (Ma et al.), suggesting that multi-strain or synbiotic formulations may provide enhanced outcomes. Taken together, the current evidence consolidates the position of *Bifidobacterium* spp. as crucial contributors to gut-brain and gut-immune homeostasis, underlining their future relevance in both clinical and functional food applications.

## Other microbial candidates

Seidler et al. provided a comprehensive review of the postbiotic potential of *Aspergillus oryzae*, which is traditionally used in East Asian food fermentation. The authors highlighted its ability to modulate the gut microbiome, enhance epithelial barrier function, influence immune responses, and impact metabolic and neural signaling. This work underscored the translational potential of fungal-derived postbiotics for gut health and related therapeutic interventions, while emphasizing the importance of standardization and quality control.

*Blautia producta* 1009924, isolated from human feces, exhibited notable probiotic potential (Chen et al.). In a DSS-induced zebrafish intestinal inflammation model, *B. producta* reduced ROS production, modulated TLR4/NF-κB signaling, decreased pro-inflammatory cytokines, and enhanced SCFA levels, thereby improving intestinal tissue integrity. Similarly, *E. casseliflavus* SHAMU-QH-02, isolated from the human biliary tract, was found to exhibit broad-spectrum antagonistic activity, antioxidant and anti-inflammatory effects, in addition to being safe for functional applications (Li et al.).

Recent genome sequencing of four human *Akkermansia* spp. isolates revealed a low genetic risk profile with limited AR and virulence genes, and functional annotations enriched in metabolic pathways. These strains were found to support gut barrier integrity, modulate host metabolism, and influence immune signaling, highlighting their potential for precision microbiome-targeted interventions in metabolic and inflammatory disorders (Lu et al.).

*Weizmannella coagulans* BC99 exhibited notable probiotic and anti-inflammatory properties (Gao et al.). In a *Caenorhabditis elegans* hyperuricemia model, it reduced uric acid and xanthine oxidase levels, decreased ROS production, and improved lifespan and motility. Mechanistically, *W. coagulans* activated the transcription factors DAF-16 and SKN-1, thereby enhancing stress-response-involved gene expression and antioxidant enzyme activity. Metabolomic analysis indicated the regulation of amino acid, glycerophospholipid, and purine metabolism. These findings support *W. coagulans* BC99 as a safe and effective candidate for managing hyperuricemia and related metabolic disturbances.

Peng et al. demonstrated that the probiotic *Bacteroides fragilis* (BF839), extensively used in China to alleviate gut microbiota dysbiosis, can enhance tumor sensitivity to immune checkpoint inhibitors (ICIs) via the activation of the cyclic GMP-AMP synthase-stimulator of interferon genes (cGAS-STING) signaling pathway, suggesting that modulation of the gut microbiota with BF839 may represent a promising strategy to improve ICI efficacy in cancer therapy.

Ma et al. reported that a synbiotic treatment combining low-, medium-, and high-dose mixed probiotics (*Bifidobacterium animalis* subsp. *lactis* XLTG11, *Lcb. paracasei* Glory LP16, and *Lpb. plantarum* CCFM8661) with oligofructose alleviated DSS-induced colitis in mice by reducing inflammation, restoring colon length, enhancing intestinal barrier integrity, and increasing gut microbiota diversity and SCFA production, with therapeutic effects dependent on the probiotic dose.

Synbiotic and multistrain formulations, combining *Lactobacillus, Bifidobacterium*, and *Enterococcus* species, demonstrated enhanced probiotic efficacy (Liao et al.; Ma et al.; Teng et al.). These combinations improved SCFA production, restored microbiota diversity, and suppressed intestinal inflammation more effectively than single strains. These synergistic interventions highlighted the translational potential of multistrain probiotics for precision microbiome modulation and for therapeutic applications in gastrointestinal health.

In addition, several recent studies, while not directly testing probiotics, provided important insights into gut microbiota modulation and host health with clear implications for probiotic research. For example, supplementation with *Portulaca oleracea* (purslane) in aging rats improved gut morphology, increased fecal SCFA levels, and shifted microbial composition by reducing Firmicutes and Fusobacteria while modulating metabolic pathways (Deng et al.). These findings suggest that dietary interventions can target microbiota composition and metabolic output in ways similar to probiotic supplementation.

Similarly, in the context of infectious diseases such as cholera, studies in this Research Topic highlighted probiotics as a potential adjunctive strategy to enhance gut barrier function, compete with pathogens, and modulate immunity, illustrating their translational potential even in settings traditionally managed by environmental or pharmacological interventions (Chowdhury et al.).

Other studies focused on functional metabolites, such as D-tryptophan, which was found to exhibit antibacterial, immunomodulatory, and anti-biofilm properties (Wang et al.), indicating that dietary or microbial-derived compounds can act synergistically with probiotics to improve host health.

Clinical evidence further supported the use of probiotics and synbiotics in treating metabolic and liver diseases. In NAFLD patients, supplementation with probiotics or synbiotics significantly reduced liver enzymes, liver stiffness, insulin resistance, and BMI, highlighting their therapeutic efficacy (Song et al.).

Finally, Mendelian randomization studies linking gut microbiota with trimethylamine-N-oxide levels underscored specific microbial taxa that increased or decreased host susceptibility to metabolic risks (Yu et al.). These mechanistic insights can inform the selection of probiotic strains aimed at modulating cardiovascular risk factors.

## Synthesis and translational Insights

The articles in this Research Topic collectively highlight the evolution of probiotic research from a mechanistic understanding to translational and industrial applications. Key themes emerge across taxonomic groups, functional effects, and intervention strategies.

## Gut barrier and immune modulation

*A. muciniphila* spp. (Lu et al.) was found to support intestinal homeostasis by enhancing epithelial integrity, reducing metabolic endotoxemia, and promoting anti-inflammatory signaling. Similarly, genera such as *Blautia producta* (Chen et al.), *W. coagulans* (Gao et al.), and *E. casseliflavus* SHAMU-QH-02 (Li et al.) were observed to exert immunoregulatory and anti-inflammatory effects through SCFA production, ROS modulation, and cytokine regulation. In addition, *B. fragilis* BF839 was extensively studied for its ability to modulate gut microbiota and enhance antitumor immunity (Peng et al.). *Lactobacillus johnsonii* showed promise in promoting digestive health by modulating immunity, enhancing gut barrier function, and maintaining microbiota balance, with future studies needed to clarify its mechanisms and provide experimental support for its therapeutic applications (Zhou et al.). In the MASH model, *Lcb. rhamnosus* GG (LGG) reduced pro-inflammatory cytokines, inhibited TGF-β/SMAD signaling, restored intestinal barrier integrity, and prevented endotoxin translocation, thereby alleviating liver inflammation and fibrosis (Wang et al.).

## Resilience and formulation advantages

Spore-forming probiotics, exemplified by *B. coagulans* LMG S-31876 (Kallur et al.), displayed high thermal stability and supported host immunity, lipid metabolism, and stress-related outcomes. This highlights their suitability for robust industrial formulations.

## Next-generation and precision microbiome interventions

Next-generation probiotics, including *A. muciniphila* (Lu et al.) and *Faecalibacterium prausnitzii* (Song et al.), provided targeted modulation of gut mucosal integrity and SCFA-driven immune balance. These approaches enabled personalized microbiome interventions for metabolic and inflammatory disorders.

## Synergistic and multistrain approaches

Synbiotic and multistrain formulations incorporating *Lactobacillus, Bifidobacterium*, and *Enterococcus* species (Liao et al.; Ma et al.; Teng et al.) enhanced SCFA production, restored microbial diversity, and achieved superior inflammation suppression compared to monostrain interventions. These findings underscore the translational advantage of synergistic formulations for precision microbiota modulation.

## Translational and industrial implications

The field is moving toward practical application, emphasizing strain safety, viability, stability, scalability, and regulatory compliance. Techniques such as encapsulation, lyophilization, and prebiotic co-formulation were found to improve functional performance and shelf stability (Liao et al.; Song et al.). Comprehensive safety profiling, as exemplified by *E. casseliflavus* SHAMU-QH-02, was deemed critical for regulatory approval (Li et al.).

In conclusion, this Research Topic consolidates mechanistic, functional, and translational insights, highlighting the potential of probiotics to shape next-generation health interventions across clinical, nutritional, and industrial domains.

